# Global service learning and health systems strengthening: An integrative literature review

**DOI:** 10.1016/j.heliyon.2018.e00713

**Published:** 2018-08-02

**Authors:** Adam Beaman, Reiko Asano, David Sibbritt, Phillip J. Newton, Patricia M. Davidson

**Affiliations:** a University of Technology Sydney, Australia; b Johns Hopkins School of Nursing, USA; c Western Sydney University, Australia

**Keywords:** Health profession, Nursing, Public health, Education

## Abstract

**Introduction:**

The sustainability of many global interventions, in the absence of adequate local financial and human resources to sustain them in the long term, is questioned. In response, there has been a shift in focus among global health actors towards the strengthening of local health systems via global service learning to effectively, efficiently and sustainably deliver healthcare and build capacity. There has been considerable research examining the benefits of global service learning experiences for students, but limited research has been conducted to determine the impact that health sciences global service learning experiences are having on the host country health systems.

**Main text:**

An integrative review of the literature was conducted to examine the linkages between global service-learning and health systems strengthening. A comprehensive search of international literature from 2005 to 2017 in PubMed, CINAHL, Embase, ERIC, Scopus and Web of Science databases was conducted. The search was limited to peer-reviewed articles published in English language. Thematic analysis revealed three key themes: a dominant service-LEARNING typology, a unidirectional pattern from high-income to low and middle-income countries, and the preeminence of the nursing discipline in global service learning. There was limited evidence on sustainability and health systems strengthening.

**Conclusions:**

The healthcare workforce of the future is willing to meet the challenges facing health systems across the globe. Global service-learning has yet to be examined in the context of health systems strengthening and particularly within a context of reciprocity. The onus is on higher education institutions in high-income countries to develop and deliver evidence-based global service-learning that is beneficial and engaging for students while most effectively meeting the need of the global community.

**What we already know:**
• There is a global focus on internationalization and the benefit of the experience of diversity for students
• The focus on health systems strengthening in global health is increasing, though efforts to date have been unbalanced
• Global service learning programs for nursing students are in high and increasing demand

**What this paper adds:**
• Global service learning programs for nursing students generally achieve beneficial educational outcomes
• There is limited consideration of impact on the host country
• There is limited consideration of health systems strengthening and capability development

## 1 Introduction

Over the last two centuries the process of global interconnectedness has intensified, leading to a number of social, political and economic drivers in the context of global health (Koplan et al., 2009). A range of factors, such as power differentials and health care disparities, have led to a range of initiatives for high income countries (HICs) to support low and middle income countries (LMICs) (Birn, 2009). At the same time, rapid technological advancements have allowed individuals in HICs to access both information illustrating the extent of these disparities, and the means to travel to LMICs with relative ease and see these disparities in person (McCall and Iltis, 2014). There has also been a recognition of the importance of cultural awareness and competence to being astute and integrated global citizens (Jogerst et al., 2015).

Effectively implementing initiatives that can improve health and healthcare in LMICs is complex and multifaceted, and motivations for international work range from a mission based agenda driven by a desire for compassion to motivations for national security (King, 2002). The aspiration to ‘learn and serve’ is a challenging issue requiring achieving clarity of nomenclature, transparency of agenda as well as shared, collaborative and negotiated models (McCall and Iltis, 2014). Community engagement pedagogies that are commonly termed ‘service-learning’, combine learning goals and community service with the motivation of achieving educational outcomes for students as well as benefits for communities (Sigmon, 1994). Whilst conceptually alluring there are a number of pitfalls and unless these are addressed systematically, what is well intentioned can have adverse effects.

### 1.1 Health systems strengthening

There can be little doubt that the impact of disease-specific interventions at the global level has had a significant effect in reducing disease prevalence, particularly in the field of infectious disease (Ozawa et al., 2016). However, in recent decades researchers have called into question the sustainability of impact of many global interventions in the absence of adequate local financial and human resources to sustain them in the long term (Biesma et al., 2009). In particular, global health organizations have identified local financial and human resource shortages as perhaps the major obstacles (Chen et al., 2004). In response, there has been a shift in focus towards the strengthening of local health systems to effectively, efficiently and sustainably deliver healthcare (Hafner and Shiffman, 2013). This is evidenced by the allocation of a combined US$1.1 billion in funding allocation by the U.S. President's Emergency Plan for AIDS Relief (PEPFAR), the Global Alliance for Vaccines and Immunization (GAVI) and the Global Fund, on HSS between 2005 and 2010 (Hafner and Shiffman, 2013).

The World Health Organization's publication “Strengthening health systems to improve health outcomes” (2007) defines health systems as “all organizations, people and actions whose primary intent is to promote, restore or maintain health.” This definition is significantly broader than those traditionally emphasizing publicly owned health systems providing services to individuals and families. In expanding upon this definition, WHO (2007) emphasizes the role of the health workforce in health systems strengthening (HSS), as navigators to help patients access care and as advocates for healthcare policy improvements. WHO (2007) provides six building blocks for HSS: service delivery; health workforce; information; medical products, vaccines and technologies; financing; and leadership/governance. WHO's definition has been widely adopted, including by PEPFAR, GAVI and the Global Fund (Hafner and Shiffman, 2013).

A review of HSS efforts in Bangladesh, Ethiopia, Kyrgyzstan, Thailand, and the Indian state of Tamil Nadu, found significant improvements in health indicators, services or policies in these countries and State when compared to neighboring countries (Balabanova et al., 2013). The study attributed these successes to solid and committed governance, effective bureaucracies and institutions, innovation, and health system resilience. An analysis of HSS expenditure found that on the whole HSS efforts have been skewed towards service delivery, human resources, and medicines and technology, and that a more balanced approach reemphasizing governance, financing and information may be warranted (Warren et al., 2013).

It should be noted though, that there has been ebbs and flows in preferences for horizontal (those engaging all elements of the local health system) versus vertical (those operating independently of the local health system) approaches to global health programming. This is due to a range of factors, including: economic and political factors in high income countries; the challenge of developing productive partnerships and achieving measurable outcomes with local health systems; and the desire to meet millennium development goals targets (Hafner and Shiffman, 2013). Other criticisms of HSS have centered on: the vagueness of the concept; the fact that many global health organizations are delivering disease-specific initiatives simply re-branded as HSS initiatives, and that local efforts have been undermined by global actors with superior resourcing (Marchal et al., 2009).

### 1.2 Service-learning

Service-learning is an experiential approach to learning based on the principle of reciprocal learning (Sigmon, 1979). The approach has its roots in the educational philosophies of John Dewey's writings on the nature of understanding and the benefits of participation (Giles and Eyler, 1994). Sigmon provided four typologies of service-learning (Sigmon, 1994):

*Service-LEARNING: Learning goals primary; service outcome secondary*

*SERVICE-learning: Service outcomes primary; learning goals secondary*

*Service-Learning: Service and learning goals completely separate*

*SERVICE-LEARNING: Service and learning goals of equal weight and each enhances the other for all participants*

Global service-learning (GSL) in the health sciences generally involve a short (one to two week) trip to a LMIC to provide a service, most often directly related to clinical care. The benefits of GSL experiences for students are well documented, and include personal growth, improved cultural competence, increased understanding of the global health environment, and improved language skills (Larson et al., 2010; Puri et al., 2013; Reuland et al., 2008; Sherraden et al., 2013). However, some researchers have questioned whether GSL programs, often non-reciproal (i.e. HIC students visiting low and middle income countries LMICs but not the reverse) and thus not truly exchanges, are of any real benefit to the host community (Kulbok et al., 2012). In response to these concerns, there has been a substantial amount of research into the core principles that should guide GSL in the health sciences in ensuring that they are of benefit to the host community. These include ensuring strong community partnerships, a commitment to reciprocity, and sustainability of funding (Crabtree, 2013; Lattanzi and Pechak, 2011; McKinnon and Fealy, 2011). However, no research has been conducted to determine the impact that health sciences GSL experiences are having on the host country health systems.

## 2 Method

**Fig. 1:**
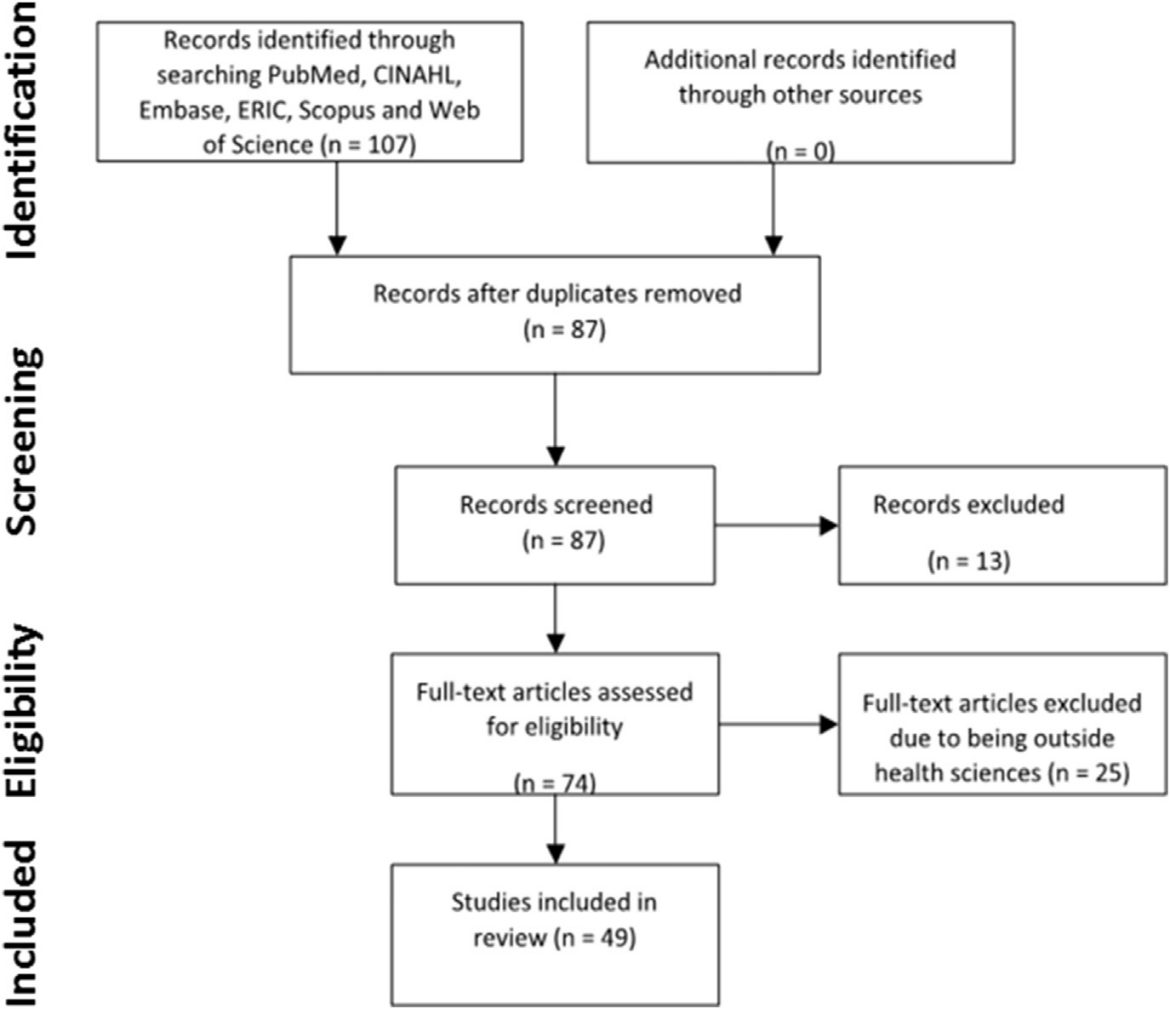
Literature search methodology utilizing PRISMA framework. The electronic data bases PubMed, CINAHL, Embase, ERIC, Scopus and Web of Science databases were searched. The searches yielded 107 results. There were 20 duplicate articles, 13 exclusions and 25 articles did not meet the inclusion criteria, leaving 49 articles for review.

The literature review followed the integrative review method, using the process problem identification, literature search, data analysis, and synthesis. The electronic data bases PubMed, CINAHL, Embase, ERIC, Scopus and Web of Science databases were searched to examine the linkages between GSL and health systems strengthening. Keywords included “*Community Health Planning*” or “*Delivery of Health Care*” or “*Health Care Economics and Organizations*” or “*health system*” or “*health systems*” or “*host community*” or “*host communities*” and “*service-learning*” and “*global”* or “*international”* or “*overseas”* or “*foreign*”. The searches yielded 107 results. There were 20 duplicate articles, 13 exclusions and 25 articles did not meet the inclusion criteria, leaving 49 articles for review. Information was retrieved using a data extraction tool and key findings analyzed using thematic analysis. PRISMA literature search methodology is graphically represented in Fig. 1.

A summary of retrieved articles is summarized in the Supplementary Table. Three themes summarizing literature findings are presented below.

## 3 Results

### 3.1 Theme 1: a dominant service-LEARNING typology

Consistent with Sigmon's service-LEARNING typology, the majority of reviewed studies focused on outcomes for students, improved cultural competence, increased understanding of the global health environment, and improved language skills (Sigmon, 1994). There were some notable exceptions; some studies also measured health outcomes in host country populations (Chaponniere et al., 2013; Downes et al., 2007), examined the intended and unintended consequences of GSL (Crabtree, 2013) and outlined principles for mutually beneficial GSL (Cipriani, 2017; Dalmida et al., 2016; Hartman et al., 2014; Kreye and Oetker-Black, 2013; Lattanzi and Pechak, 2011; Logar et al., 2015; McKinnon and Fealy, 2011).

### 3.2 Theme 2: a unidirectional pattern

The studies reviewed primarily described partnerships with health facilities within LMICs, the stated purpose of which was to host visiting students and faculty from LMICs, primarily the U.S. While studies noted the importance of reciprocity to ethical and effective GSL (Cipriani, 2017; Dalmida et al., 2016), among case studies there was limited discussion of students in LMICs visiting HICs for reciprocal experiences.

### 3.3 Theme 3: the preeminence of nursing

While the literature search methodology included all students of the health sciences, it is evident from the search that within the health sciences the concept of global service-learning is most often associated with the nursing discipline. Other disciplines included engineering and education (Chaponniere et al., 2013), medicine (Chuang et al., 2015; Myers and Fredrick, 2017), pharmacy (Chuang et al., 2015; Davis et al., 2015; Footer et al., 2015), speech pathology (Colodny et al., 2014), physical therapy (Footer et al., 2015; Pascal, 2011), dentistry (Kaddoura et al., 2014; Puri et al., 2013), audiology (Krishnan et al., 2016). Many of these studies were also interdisciplinary in nature, involving students from two or more of the aforementioned disciplines.

## 4 Conclusions

The literature review conducted has highlighted that the primary aims of the majority of GSL experiences are student development and to a lesser extent host community health, consistent with Sigmon's service-LEARNING typology (Sigmon, 1994). GSL has yet to be examined in the context of HSS and particularly within a context of reciprocity. This is perhaps due to the nature of nursing education; educators and students alike may feel compelled to develop their clinical skills as part of their education. Indeed, in order to meet accreditation requirements in pre-licensure programs, this is oftentimes mandatory. But as the discipline of global health evolves, a re-consideration of GSL experiences appears warranted. Moreover, it can be argued that efforts to deliver GSL should be holistic and incorporate HSS endeavors that are outside the realm of the traditional clinical service GSL model.

Anecdotally, there is a large demand among students of the health sciences for GSL, and as a consequence, an increased emphasis on competency development (Jogerst et al., 2015). Implicitly, if this demand outstrips the supply of appropriate and viable GSL opportunities, then there is a large and willing temporary workforce that is being underutilized and that could have benefit to the global community. Considering students wishing to undertake GSL in this way, through an organizational lens and as a part of the global health workforce, may be useful in the context of this research. The use of the word “temporary” here is also key, as it is a challenge that GSL educators have been wrestling with for decades; how can truly sustainable improvements occur when the workforce is subject to constant turnover?

An additional issue to consider, particularly in light of the Sustainable Development Goals, is the affordability and adverse environmental impact of air travel. Traditional GSL experiences have typically involved in-person travel to a community in a developing country (Kulbok et al., 2012). But GSL that can be delivered without travel may be increasingly possible as local, community-based organizations serve immigrant populations, and technological advancements continue to shape the learning environment.

Alternative approaches to GSL may make it possible for experiences to be viable for all students, regardless of their financial means. Indeed, higher education institutions have a moral obligation to deliver the same quality of educational experience to all admitted students. Moreover, such an approach could also ease financial pressure on educational institutions covering some of the cost of traditional GSL, and make a contribution to reducing carbon emissions.

**Fig. 2:**
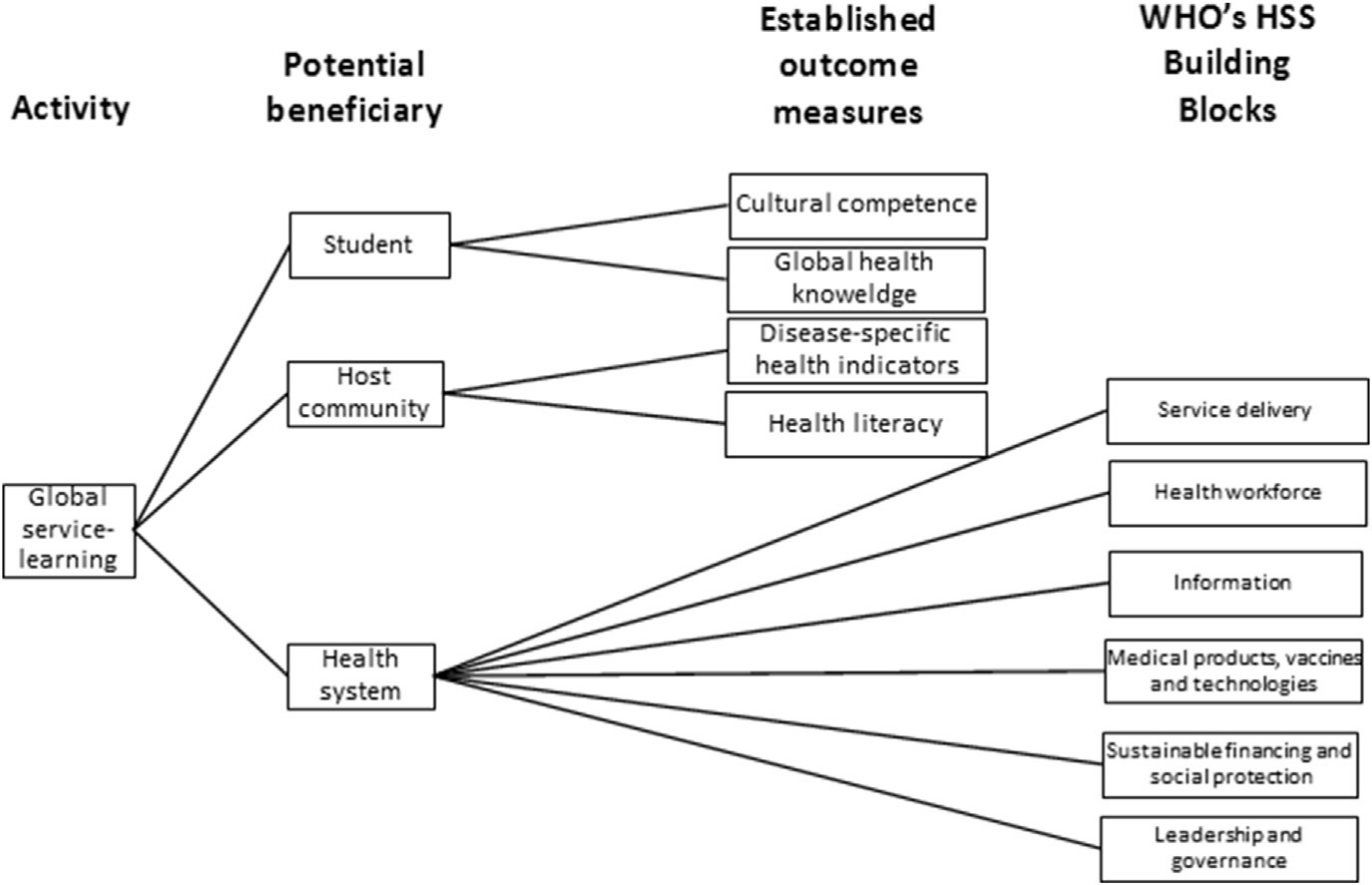
Conceptual model. Demonstrates the relationship between student, host community and health system.

A paper identifying interprofessional global health competencies for health professionals (Jogerst et al., 2015) has particular relevance to the future of GSL. The paper lists 11 domains, which would ideally be considered in GSL program development, in addition to the more traditional educational aims of cultural competence and global health awareness. Moreover, these competencies have considerable overlap with WHO's (2007) six HSS building blocks, and their consideration may also broaden student understanding of the nature of health systems and their impact on individual and community health. Fig. 2 provides a conceptual model demonstrating the relationship between student, host community and health system. Deeper consideration of these factors may lead to more thoughtful and intentional planning of health care services.

An investigation of expenditure in HSS found that service delivery, human resources, and medicines and technology, have been emphasized over governance, financing and information (Warren et al., 2013). One particular area of opportunity for nursing student GSL may be contributing to the improvement of health information systems. For example, in countries that have the infrastructure to support internet connections and electronic data collection, students may be able to remotely assist in the analysis, dissemination and use of these data. This is an activity that has the potential for sustainable, measurable impact, while simultaneously being financially viable for students and HIC universities.

As recent events like the Ebola and Zika outbreaks and the ongoing global tensions surrounding immigration policy have demonstrated, communities, countries and global regions do not operate in isolation. This will undoubtedly fuel interest in the global health discipline, particularly as technological advancements increasingly allow for instant access to information from across the globe. And it appears that the healthcare workforce of the future is willing to meet this challenge. The onus is on higher education institutions in HICs to develop and deliver evidence-based GSL that is beneficial and engaging for students while most effectively meeting the need of the global community.

## Declarations

### Author contribution statement

All authors listed have significantly contributed to the development and the writing of this article.

### Funding statement

This research did not receive any specific grant from funding agencies in the public, commercial, or not-for-profit sectors.

### Competing interest statement

The authors declare no conflict of interest.

### Additional information

No additional information is available for this paper.
