# Supplementary material for: Global service learning and health systems strengthening: An integrative literature review
Source: Heliyon. 2018 Aug 2;4(8):e00713. doi: 10.1016/j.heliyon.2018.e00713 (PMC6082917; doi:10.1016/j.heliyon.2018.e00713)
Supplement: Supplementary Table [file mmc1.docx]

| **Authors and year** | **Title** | **Country/ Experience/ Purpose** | **Design/ Method/ Models** | **Sample/ Size** | **Findings** |
| --- | --- | --- | --- | --- | --- |
| (Abedini, Gruppen, Kolars, & Kumagai, 2012) | Understanding the effects of short-term international service-learning trips on medical students | Authors from: U.S. Type of Experience: One-week international service-learning trips (ISLTs); One way U.S. to Cuba, the Dominican Republic, Guatemala, Jamaica, or Peru Purpose: To understand what meaning(s) preclinical students attributed to participation in one-week international service-learning trips | A 15-item online questionnaire, Face-to-face, semi structured interviews | A random sample of 24 students; 17 students agreed to participate | Students reported:  --improved clinical skills and outcomes for the community  --a range of challenges associated with the nature of the visit and working in a low-income setting  --improved understanding of working in a global health context |
| (Amerson, 2010) | The impact of service-learning on cultural competence | Authors from: U.S. Type of Experience: A week-long immersion experience in Guatemala Purpose: To evaluate the self-perceive cultural competence of baccalaureate nursing students enrolled in a community health nursing course following the completion of service-learning projects with local and international communities | Survey; The Transcultural Self-Efficacy Tool (TSET) was administered at the beginning and completion of the semester. | A convenience sample of 69 baccalaureate nursing students enrolled in a community health nursing course | Nursing students perceived an increase in their abilities in cognitive, practical, and affective dimensions following participation in a service-learning project |
| (Amerson & Livingston, 2014) | Reflexive photography: an alternative method for documenting the learning process of cultural competence | Authors from: U.S. Type of Experience: One-way trip to Guatemala Purpose: To evaluate the learning process of cultural competence during an international service-learning project in Guatemala | Qualitative descriptive study; conducted family and community assessments, engaged in home visits, and provided health education | A purposive sample of 10 baccalaureate nursing students traveled to Guatemala | Making home visits and teaching others from a different culture increased students' transcultural self-efficacy |
| (Bentley & Ellison, 2007) | Increasing Cultural Competence in Nursing through International Service-Learning Experiences | Authors from: U.S. Type of Experience: One-way international service-learning trip to Ecuador Purpose: To prepare nursing students to become culturally competent practitioners | Descriptive study; student evaluation of course and trip | 25 nursing students | The trip and immersion experience led to changed attitudes toward working with those from other cultures. --Tailored cross-cultural courses help increase knowledge about other cultures including knowledge about values, beliefs, and practices --For most students to achieve greater cultural competence, immersion experience was needed --In an immersion experience, like an international trip, students may more easily leave their daily personal and professional relationships and responsibilities behind and truly experience the lives and environment of those visited --The specific course and service-learning international trip made a valuable difference in the students' development of cultural competence |
| (Booth, 2016) | Compassion: a universal language | Authors from: U.S. Type of Experience: One-way medical trip to Indochina Purpose: To utilize any available resources in nursing education to demonstrate and model compassionate behaviors in order for students to not lose sight of what compassion means for the profession and calling of nursing | Descriptive study | N/M | Students perceived that despite cultural differences, language barriers, and thousands of miles of separation, compassion had the capacity to reduce these barriers |
| (Chaponniere et al., 2013) | Measuring the impact of health education modules in Cameroon, West Africa | Authors from: U.S. Type of Experience: One-way trip to Cameroon to create a sustainable source of clean water and improve the overall health of people in Nkuv Purpose: To evaluate the impact of a health education program in a small rural community in Cameroon | Descriptive study | Engineering, nursing, and education students at Hope College | --Reduced incidence of childhood diarrhea  --Students learned the importance of pairing service-learning and research that respect local cultures to foster a healthier global community |
| (Chuang et al., 2015) | Medical and pharmacy student concerns about participating on international service-learning trips | Authors from: U.S. Type of Experience: One-way international service-learning trips (ISLT) Purpose: To identify student concerns before and after attending a service-learning trip and the impact on student satisfaction and achievement of personal and professional goals | Literature review of pre and post surveys | 35 medical and pharmacy students who attended ISLTs | --Decrease in concerns related to cultural barriers, disease/epidemics, natural disasters, terrorism, travel monetary issues, hospitality and food --Languages and group dynamics still remained concerns post-trip |
| (Cipriani, 2017) | Integration of International Service-learning in Developing Countries within Occupational Therapy Education: Process and Implications | Authors from: U.S. Type of Experience: N/A Purpose: N/A | Literature review | N/A | The following seven issues were emphasized as to mutually beneficial GSL:  1. Occupational justice  2. Fidelity to accreditations standards 3. Reciprocal school-community partnerships and learning  4. A focus on safety  5. Cultural competence  6. Guided self-reflection  7. Sustainability |
| (Colodny et al., 2014) | The development of a feeding, swallowing and oral care program using the precede-proceed model in an orphanage-hospital in Guatemala | Authors from: U.S. Type of Experience: N/A Purpose: To evaluate a long-term on-going international academic service-learning (I-ASL) intervention | A quantitative study, using the PRECEDE-PROCEED model | 56 speech language pathology graduate students | The researchers perceived a trusting and mutually supportive relationship was built between the I-ASL team and the host organization |
| (Crabtree, 2013) | The Intended and Unintended Consequences of International Service-Learning | Authors from: U.S. Type of Experience: N/A Purpose: To examine issues related to university-community engagement in global contexts, particularly consequences for host communities | Case study | N/A | Outcomes vary by intended and unintended, positive and negative, for students, faculty members, organizations and their staff, and the communities that host visiting teams from U.S. universities |
| (Curtin, Martins, & Schwartz-Barcott, 2015) | A mixed methods evaluation of an international service-learning program in the Dominican Republic | Authors from: U.S. Type of Experience: One-way trip to Dominican Republic Purpose: To examine the impact of an international service-learning experience (ISL) | Descriptive study  quantitative and qualitative | 11 female, Caucasian, senior and junior baccalaureate nursing students | Students reported: -- Increased motivation to learn and ability to speak another language. -- Enhanced understanding of the U.S. culture |
| (Curtin, Martins, Schwartz-Barcott, DiMaria, & Ogando, 2015) | Exploring the Use of Critical Reflective Inquiry With Nursing Students Participating in an International Service-Learning Experience | Authors from: U.S. Type of Experience: One-way 2-week trip to Dominican Republic Purpose: To explore the value of critical reflective inquiry (CRI) in guiding nursing students reflection on an international service-learning experience in the Dominican Republic | Qualitative descriptive study | 5 senior and 2 junior female baccalaureate nursing students | The CRI model was helpful in promoting in-depth description and reflection on the students' underlying assumptions and values, as well as identifying beginning strategies from emancipation in specific patient care situations |
| (Dalmida et al., 2016) | Volunteer Service and Service-learning: Opportunities, Partnerships, and United Nations Millennium Development Goals | Authors from: U.S., Botswana, West Indies Type of Experience: N/A Purpose: To explore approaches to service involvement and provide direction to nurse leaders and others who wish to begin or further develop global service or service-learning projects | Literature review | N/A | Before engaging in service, volunteers should consider the types of service engagement, as well as the design of projects to include collaboration, bi-directionality, sustainability, equitable partnerships, and consideration of the United Nations Sustainable Development Goals |
| (Davis et al., 2015) | Interprofessional global service-learning: A pharmacy and nursing practice experience in Botswana | Authors from: U.S. Type of Experience: 27 days Interprofessional activities in Kanye, Botswana  Purpose: To develop, implement, and assess an interprofessional global service-learning experience in rural government-run clinics and a community hospital for pharmacy and nursing students | Qualitative survey | 5 Pharmacy students 11 nursing students | Global service-learning is a valuable opportunity to prepare collaborative teamwork among pharmacy and nursing students: --Partnerships with other health care programs' established educational experiences can facilitate pharmacy program expansion into global interprofessional practice experiences |
| (Dharamsi et al., 2010) | Enhancing medical students' conceptions of the CanMEDS Health Advocate Role through international service-learning and critical reflection: a phenomenological study | Authors from: Canada Type of Experience: One-way 8 week-trip to Uganda Purpose: To explore the international service-learning (ISL) experience of three medical students and the value of critical reflection as a pedagogical approach to enhance medical students' conceptions of the Canadian Medical Education Directions for Specialists (CanMEDS) Health Advocate Role | In-depth phenomenological study | 3 medical students | Students noted: -- An increasingly meaningful sense of what it means to be vulnerable and marginalized -- A heightened level of awareness of the social determinants of health -- The related importance of community engagement -- A deeper appreciation of the health advocate role and key concepts embedded within it |
| (Downes et al., 2007) | The use of Service-Learning in drought response by universities in Ethiopia | Authors from: U.S. Type of Experience:  Purpose: To describe a successful Service-Learning experience and its outcomes that affected over 10 million Ethiopians | Descriptive study | 2,191 health science students and 350 university instructors | Service-Learning has potential benefits for various stakeholders and student. Roles included: -- Provision of clinical, direct patient care for episodic problems -- Nutritional interventions of therapeutic and supplementary feedings Implementation of preventive and promotive health activities -- Participation in the control of disease outbreaks -- On-the-job training of professionals and others in the deployment areas |
| (Footer et al., 2015) | Return on investment of international immersion programs: Stakeholder perspectives | Authors from: U.S. Type of Experience: One-way international global health immersion and service-learning trip Purpose: To develop an approach to evaluate the return on investment of international immersion programs in higher education | Mixed methods design | 13 graduate students in physical therapy, nursing, and pharmacy programs | All participants indicted: -- Increased awareness and understanding of concepts related to topics such as social determinants of health, community development, global interdependence -- Personal and academic challenges upon return home from the program related to personal beliefs and values, cultural awareness and understanding, and impact of service-learning on themselves and others |
| (Foster, Guisinger, Graham, Hutchcraft, & Salmon, 2010) | Global Government Health Partners' Forum 2006: eighteen months later | Authors from: U.S. Type of Experience: Government chief nursing officers and chief medical officers in a joint learning and planning experience  Purpose: To evaluate the outcomes of the Global Government Health Prtners (CGHP) forum | Descriptive study | 21 CGHP participants | Improvements made in human resource among participant countries included: --revising key job descriptions --improving incentives --better distribution of nurses to rural areas --continuing education and increasing the number of nurse trainers Work that remains to be done: --Policy recommendations are currently overlooked --CNOs do not have a voice in the government commensurate with their responsibilities |
| (Gates, Fletcher, Ruíz-Tolento, Goble, & Velloso, 2014) | A Pesar De Las Fronteras/"In Spite of the Boundaries": Exploring Solidarity in the Context of International Service Immersion | Authors from: U.S. Type of Experience: One-way 3-week service-learning immersion trip to Nicaragua Purpose: To learn about experiences of Nicaraguans who hosted U.S. American students in their homes and communities | Ethnographic: observation and in-depth interviews | 26 interviews with a total of 29 Nicaraguans who hosted U.S. students | Students identified cultural difference and inequality as salient themes, and that thy shaped the relationships and possibilities for joint action between U.S. students and their hosts |
| (Green, Comer, Elliott, & Neubrander, 2011) | Exploring the value of an international service-learning experience in Honduras | Authors from: U.S. Type of Experience: One-way volunteer medical mission to the isolated and mountainous western region of Honduras Purpose: To determine the effect of an international service-learning experience in Honduras on the cultural competence of the participants | Pretest-posttest Quantitative: questionnaire Qualitative: interview | 15 students | The value that was ascribed to the experienced included: -- stepping outside their world --connecting with culturally different people --awe of the community --learning innovation |
| (Hartman et al., 2014) | Fair Trade Learning: Ethical standards for community-engaged international volunteer tourism | Authors from: U.S. Type of Experience: International volunteer tourism programs operating at the nexus of university-community engagement Purpose: To articulate a set of ethical standards for international volunteer tourism | Comprehensive review | N/A | Student learning and community goals must reinforce and inform one another. Either is undermined by the absence of the other. |
| (Hope, 2008) | The development of a medical service-learning study-away program | Authors from: U.S.  Type of Experience: One-way service trip to Ecuador Purpose: the considerations for planning a study-away experience for nurses and nursing students in another country. | Summary article | 2 faculty members 3 premed students 1 MSN student 2 BSN students 10 nursing students | After the trip, some students changed their academic majors, and several made major health behavior changes. |
| (Johanson, 2009) | Service-learning: deepening students' commitment to serve | Authors from: U.S. Type of Experience: One-way service-learning trip to Mexico (9-day trip) Purpose: To emerge from the experience with enhanced cultural sensitivity | Summary article | Nursing students | All the students in the first group and the three cohorts to follow:  -- commented that seeing this level of need and sensing their ability to make a difference would have an impact on the way they practiced nursing. --their general perception was that they would be more empathic professionals and continue to volunteer and serve when they became registered nurses |
| (Jones, Ivanov, Wallace, & VonCannon, 2010) | Global service-learning project influences culturally sensitive care | Authors from: US Type of Experience: One-way service-learning trip to a small town in Russia Purpose: to examine the prevalence of components of the metabolic syndrome | Pretest-posttest | Nursing students | Students reported increased awareness of cultural differences and social determinants of health |
| (Kaddoura et al., 2014) | International academic service-learning: lessons learned from students' travel experiences of diverse cultural and health care practices in Morocco | Authors from: U.S. Type of Experience: One-way academic service-learning to Morocco Purpose: To engage students in meaningful hands-on activities to serve community-based needs | Summary article | 9 health professions students | Participants perceived that international ASL provided them with lessons in access to care, practice, interdisciplinary teamwork, communication, ethical awareness, leadership, and cultural sensitivity |
| (Kent-Wilkinson, Dietrich Leurer, Luimes, Ferguson, & Murray, 2015) | Studying abroad: Exploring factors influencing nursing students' decisions to apply for clinical placements in international settings | Authors from: Canada Type of Experience: Purpose: To explore factors influencing nursing students' decisions to study abroad | A descriptive longitudinal design using online survey | A total of 1058 nursing students registered in the 2013-2014 academic year | Students indicated that their interest in study abroad international experiences was high, with many perceived benefits, but barriers to participation were also high for these students. Financial barriers topped the list followed by family responsibilities and job obligations |
| (Kohlbry, 2016) | The Impact of International Service-Learning on Nursing Students' Cultural Competency | Authors from: U.S. Type of Experience: Purpose: The effect of and international immersion service-learning project on the level and components of cultural competence of BSN nursing students | A triangulated methodology -- the Inventory for Assessing the Process of Cultural Competence Among Healthcare Professionals-Students Version -- Cultural Self-Efficacy scale -- A demographic survey -- Open-ended qualitative questions | 121 BSN nursing students from three southern California universities | The international service-learning experiences: -- strengthen the process of becoming culturally competent -- impact and improve cultural competency |
| (Kreye & Oetker-Black, 2013) | A global service-learning experience for nursing students in Tanzania: a model for collaboration | Authors from: U.S. Type of Experience: One-way trip to Moshi, Tanzania for the 3-week service-learning experience Purpose: To address a model for creating a short-term global service-learning program | Analysis article | 2 faculty 13 junior level nursing students 1 student affairs representative | International academic partnerships, developed in accordance with WHO standards, will enhance educational experiences for nursing students both in the U.S. and abroad |
| (Krishnan et al., 2016) | Outcomes of an International Audiology Service-Learning Study-Abroad Program | Authors from: U.S. Type of Experience: One-way study abroad program to Zambia Purpose: To evaluate students' academic and civic learning, with particular interest in cultural competence, gained through participation in the Speech, Language, and Hearing Sciences in Zambia study-abroad program | Pre- and post-program administration of the Public Affairs Scale to measure changes in participants' civic learning Qualitative data included journals, end-of-program reflection papers, videos, and researcher field notes | 12 female students | Comparison of the pre- and post-program Public Affairs Scale data showed: -- a significant increase in cultural competence and a marginal increase in community engagement at the conclusion of the program  Qualitative data showed: -- participants' cultural awareness was increased, they benefited from hands-on learning, and they experienced a variety of emotions and emotional and personal growth |
| (Larkin, 2015) | Close encounters of the other kind: Ethical relationship formation and international service-learning education | Authors from: Canada Type of Experience: One-way ISL trips to Tanzania Purpose: The possibilities for a practice of ISL education that centers on the formation of socially ethical engagements with others | Analysis article | N/A | Racial and socio-economic differences are two key tensions that emerged as participants and community partners struggled to understand their respective roles |
| (Larson, Ott, & Miles, 2010) | International cultural immersion: en vivo reflections in cultural competence | Authors from: U.S. Type of Experience: One-way trip to Guatemala for an international cultural immersion course Purpose: To explore the impact of cultural immersion on students nurses' cultural competence | Qualitative descriptive study, in-depth interviews and en vivo reflective journals | 13 junior and senior nursing students | Exercises in participant-observation and reflective writing could enhance student self-awareness and their ability to benefit from a cultural immersion course |
| (Lattanzi & Pechak, 2011) | A conceptual framework for international service-learning course planning: promoting a foundation for ethical practice in the physical therapy (PT) and occupational therapy (OT) professions | Authors from: U.S. Type of Experience:  Purpose: To explore the current literature related to PT and OT ILS and build a conceptual framework for international service learning (ISL) course planning | Literature review | N/A | The five elements were identified as essential to promote a foundation for ethical practice as framed by beneficence and non-maleficence across all stakeholders in ISL: -- Cultural competency training -- Communication -- Coordination with community -- Comprehensive assessment -- Strategic planning |
| (Loewenson & Hunt, 2011) | Transforming attitudes of nursing students: evaluating a service-learning experience | Authors from: Type of Experience:  Purpose: to examine nursing students' attitudes toward homelessness before and after participation in a service-learning clinical rotation with families experiencing homelessness | Pretest-posttest intervention study | 23 students enrolled in a public health nursing course | The clinical experiences positively influenced students' attitudes and supported the value of integrating service-learning clinical opportunities with homeless individuals into nursing curricula |
| (Logar et al., 2015) | Teaching corner: "first do no harm": teaching global health ethics to medical trainees through experiential learning | Authors from: U.S. Type of Experience: N/A Purpose: There have been some attempts in the last few years to incorporate more substantive ethics guidelines into pre-departure training; however, theses tend to be too broad and often leave the trainees with very little practical guidance | Analysis article | N/A | The tension between the benefits of service-learning on the one hand and the respect for patients' rights and well-being on the other could be resolved by the application of a simulation based approach to global health ethics education |
| (Long, 2014) | Influence of international service-learning on nursing student self-efficacy toward cultural competence | Authors from: U.S. Type of Experience: One-way 2-week service-learning medical experience to Belize, Central America Purpose: measure pre-intervention scores of cultural competence and self-efficacy in nursing students; compare post-intervention scores; evaluate the effectiveness of an international service-learning experience as a teaching strategy in strengthening self-efficacy toward developing cultural competence | Quantitative study, using the Cultural Self-Efficacy Scale  Qualitative study, using the self-reflection journals | 34 students (17 in an international intervention group, 17 in a control group) | Significantly improved self-efficacy, self-confidence, skills, and self-awareness among students in working with the Hispanic culture and developing cultural competence. |
| (McKinnon & Fealy, 2011) | Core principles for developing global service-learning programs in nursing | Authors from: U.S. Type of Experience: N/A Purpose: To outline and discuss core principles for global service-learning in nursing | Analysis article | N/A | The seven key principles that can usefully guide global service-learning are: compassion, curiosity, courage, collaboration, creativity, capacity building, and competence |
| (Myers & Fredrick, 2017) | Team Investment and Longitudinal Relationships: An Innovative Global Health Education Model | Authors from: U.S. Type of Experience: A four-year Global Health Scholars Program to participate in two separate month long trips abroad  Purpose: Assessing the global health programs among medical schools | Analysis study | 191 medical students | The response from students was positive, but logistical challenges were evident including sustaining team investment and maintaining longitudinal relationships between student teams and host communities |
| (Parsi & List, 2008) | Preparing medical students for the world: service-learning and global health justice. | Authors from: U.S. Type of Experience: N/A Purpose: To discuss the growth of international service-learning in undergraduate medical education | Analysis study | N/A | Students engaged in service-learning in some of the most impoverished places experience a burden of knowledge that for many demands a response and unparalleled opportunity to address disparities. -- Medical educators who can further empower these students through training in critical reflection and response stand to inspire a broader movement for more justice in global health |
| (Pascal, 2011) | Incorporation of core values and mentoring to enhance service-learning in entry-level and post-graduate physical therapy curricula | Authors from: U.S.  Type of Experience: One way service-learning trip to Guyana Purpose: Effect of having students at different levels of education and clinical experience work together internationally provides a unique opportunity for peer teaching and direct application of the core values of physical therapy professionalism. | Qualitative study, using questionnaires and reflection paper | 5 physical therapy students | Service-learning can be an effective tool to promote learning and awareness of global health issues at the entry-level and post-graduate levels |
| (Puri et al., 2013) | Student perception of travel service-learning experience in Morocco | Authors from: U.S. Type of Experience: One-way service-learning trip to Morocco Purpose: To explore the perceptions of health profession students participating in academic service-learning in Morocco with respect to adapting health are practices to cultural diversity | Semi-structured, open-ended interviews | 9 dental hygiene and nursing students | Students perceived growth as a health care providers. The application of knowledge and skills learned in academic programs and service-learning settings were found to help in bridging the theory-practice gap |
| (Richards & Novak, 2010) | From Biloxi to Cape Town: Curricular Integration of Service-learning | Authors from: U.S. Type of Experience: One-way service-learning trip to Cape Town, South Africa Purpose: to review the service-learning framework, course planning, and implementation of a recent service-learning project | Review article | 4 senior nursing students and 1 science student | students held a favorable view of service-learning and understood the components involved in its process |
| (Ryan-Krause, 2016) | Short-Term Global Experiences: Reflections, Themes, and Implications | Authors from: U.S. Type of Experience: One-way service-learning trips to Central America and the Caribbean Purpose: to articulate the essential les- sons learned from short-term experiences and to offer directions for planning and executing more meaningful and long-lasting outcomes for nursing students and host communities | Qualitative study, using post-travel reflection paper | 89 advanced practice nursing students | Improvements were noted in clinical skills and linguistic abilities. Challenges included ethical issues of undermining the value of local providers, obstacles to appropriate follow-up care, and disruption of community life. |
| (Saffran, 2013) | Dancing through Cape Coast: ethical and practical considerations for health-related service-learning programs | Authors from: U.S. Type of Experience: One-way service-learning trip to Ghana Purpose: to explore some of the ethical and practical issues that arise when U.S. students work in health-related programs in developing countries | Commentary article | N/A | Service-learning programs should lead students to consider issues such as which basic services people are entitled to, regardless of where and in what circumstances they live, and how differences in access to social and economic resources contribute to health disparities on a global scale. |
| (Sherraden, Lough, & Bopp, 2013) | Students Serving Abroad: A Framework for Inquiry | Authors from: U.S. Type of Experience: N/A Purpose: To propose a framework for inquiry on international service programs | Essay | N/A | Differences in outcomes for students, host communities, and home colleges and universities are the result of variations in individual and institutional characteristics and service activities. |
| (Smit & Tremethick, 2014) | Preceptorship in an international setting: Honduran nurses and American nursing working together | Authors from: U.S. Type of Experience: One-way international service-learning trip to Honduras Purpose: to describe the anticipated rewards and barriers of Honduran nurses working as preceptors for students from the United States and to describe the experiences of Honduran nurse preceptors and students working together in a service-learning experience in Honduras | A qualitative descriptive design | Group 1: A convenience sample, 15 nurses  Group 2: A purposive sample of 3 of the 10 nurses Group 3: A purposive sample of 8 of the 9 nursing students | Nurse preceptors in Honduras were receptive to working with nursing students from the United States and felt that sharing knowledge with the students was the primary re- ward for their preceptorship.  Students valued the opportunity to practice skills in a supportive environment with receptive patients. |
| (Stoner, Perry, Wadsworth, Stoner, & Tarrant, 2014) | Global citizenship is key to securing global health: the role of higher education | Authors from: U.S., New Zealand Type of Experience: N/A Purpose: To argue that through consciously identifying global health within the constructs of global citizenship, institutions of higher education can play an instrumental role in fostering civically engaged students capable of driving social change. | Review article | N/A | 1. Personal health is not solely an individual, self-serving act; rather, the consequences of our lifestyle behaviors have deep and wide consequences extending to the community, national, and global contexts.  2. Being a true global citizen means one is: (a) cognizant of these interconnections and the role personal decisions play in each context; and (b) civically engaged and capable of driving social change. |
| (Tremethick & Smit, 2014) | Honduran Nurses' Work-Related Rewards and Challenges: Implications for International Service-learning and Collaboration | Authors from: U.S. Type of Experience: Service-learning experience in Honduras Purpose: To describe the current status of healthcare in Honduras and their study to learn about the work of Honduran nurses | A qualitative study, using semi-structured interviews, open-ended questions | Convenience sampling to interview 15 nurses | --Nurses reported that their perceived rewards were centered on caring and helping others. --Negative experiences and dissatisfaction with the lack of resources for healthcare services reported by Honduran nurses --Whether working with Honduran nurses in a mission setting or with students in a study abroad program, it is vital to understand the high demands on Honduran nurses of the day-to-day work. --Assisting these local nurses in their educational advancement would help to establish mutually beneficial partnerships by giving back to them. |
| (Ventres & Wilson, 2015) | Beyond ethical and curricular guidelines in global health: attitudinal development on international service-learning trips | Authors from: U.S., El Salvador Type of Experience: International service-learning trips in low and low/middle income countries Purpose: To explore how attitudes contribute to the success or failure of international service-learning trips | Review article | N/A | 1. The attitudes with which visiting health professionals approach international service-learning activities can be important components of the success or failure of the experiences.  2. Through thoughtful consideration of attitudes and approaches, participants on such trips can build a framework for rich and rewarding experiences in global medicine and global health |
| (Watzak, Engelhardt, Bentley, & Self, 2015) | Assessment of ethics and values during an interprofessional, international service-learning experience | Authors from: U.S. Type of Experience: Multi-year, short-term medical service-learning trip to Bolivia Purpose: to identify and review ethical issues that arose during the trip | Post-trip survey and pre- and post-trip journaling prompts | 30 participants including faculty and second through  fourth-year students enrolled in medical, nursing,  pharmacy, public health, and dental programs | --Team members felt they acted ethically.  --Pre-trip planning processes are required to be reviewed.  --Uncertainty in novel situations was the major cause of anxiety for participants |
| (Wittmann-Price, Anselmi, & Espinal, 2010) | Creating opportunities for successful international student service-learning experiences | Authors from: U.S.  Type of Experience: N/A Purpose: To extract lessons learned from an actual trip and dissects them from a safety, legal, and educational standpoint. | A qualitative study, using reflective journals | N/A | Service-learning experiences are educational events; as such, faculty benefit from comprehensive planning and preparation to maximize the trip’s focus on achieving the learning outcomes. |
